# Application of Molecular Modeling to Development of New Factor Xa Inhibitors

**DOI:** 10.1155/2015/120802

**Published:** 2015-09-21

**Authors:** Vladimir B. Sulimov, Irina V. Gribkova, Maria P. Kochugaeva, Ekaterina V. Katkova, Alexey V. Sulimov, Danil C. Kutov, Khidmet S. Shikhaliev, Svetlana M. Medvedeva, Michael Yu. Krysin, Elena I. Sinauridze, Fazoil I. Ataullakhanov

**Affiliations:** ^1^Research Computer Center, Moscow State University, Leninskie Gory 1, Building 4, Moscow 119991, Russia; ^2^Dimonta, Ltd., Nagornaya Street 15, Building 8, Moscow 117186, Russia; ^3^Laboratory of Physical Biochemistry, National Research Center for Hematology, Russian Academy of Medical Sciences, Novyi Zykovskiy Proezd, 4a, Moscow 125167, Russia; ^4^Laboratory of Biophysics and Physiology of Cell, Center for Theoretical Problems of Physicochemical Pharmacology, Russian Academy of Sciences, Kosygin Street, 4, Moscow 119991, Russia; ^5^Faculty of Chemistry, Moscow State University, Leninskie Gory 1, Building 3, Moscow 119991, Russia; ^6^Faculty of Chemistry, Voronezh State University, Universitetskaya Plóshchaď 1, Voronezh 394006, Russia; ^7^Federal Research and Clinical Center of Pediatric Hematology, Oncology and Immunology, Samory Mashela Street 1, Moscow 117198, Russia; ^8^Faculty of Physics, Moscow State University, Leninskie Gory 1, Building 2, Moscow 119991, Russia

## Abstract

In consequence of the key role of factor Xa in the clotting cascade and absence of its activity in the processes that do not affect coagulation, this protein is an attractive target for development of new blood coagulation inhibitors. Factor Xa is more effective and convenient target for creation of anticoagulants than thrombin, inhibition of which may cause some side effects. This study is aimed at finding new inhibitors of factor Xa by molecular computer modeling including docking SOL and postdocking optimization DISCORE programs. After validation of molecular modeling methods on well-known factor Xa inhibitors the virtual screening of NCI Diversity and Voronezh State University databases of ready-made low molecular weight species has been carried out. Seventeen compounds selected on the basis of modeling results have been tested experimentally *in vitro*. It has been found that 12 of them showed activity against factor Xa (IC_50_ = 1.8–40 *μ*M). Based on analysis of the results, the new original compound was synthesized and experimentally verified. It shows activity against factor Xa with IC_50_ value of 0.7 *μ*M.

## 1. Introduction: Role of Factor Xa in the Body and Structure and Known Inhibitors

Thromboembolic diseases including deep vein thrombosis, myocardial infarction, and thromboembolism of the pulmonary artery are the most widespread cases of death in Europe and North America [1]. Traditionally the researches of anticoagulant have been focused on the development of thrombin inhibitors which have a great number of disadvantages [2]. Recent studies have shown that factor Xa enzyme acts on the basic components of the clotting cascade by turning prothrombin into thrombin catalyzing the reaction of fibrin formation. Thereby factor Xa may be more convenient target for design of new anticoagulants. The fact of the matter is that serine protease factor Xa is the main enzyme in the coagulation cascade, and it is a connecting link between internal and external ways of blood coagulation activation. Also coagulation and inflammation are the only known factor Xa functions, whereas thrombin is more polyfunctional. Besides coagulation role thrombin plays anticoagulation role and anti-inflammation role by protein C activation and can initiate the cell proliferation and migration [3], so thrombin inhibition may result in undesirable effect. Factor Xa inhibitors do not cause the rebound effect in contrast to thrombin [4]. Furthermore the coagulation cascade is organized so that one molecule of factor Xa generates a lot of thrombin molecules [5]. Therefore factor Xa inhibition may be more efficient than the thrombin inhibition [3, 6]. It is conceivable that inhibition of factor Xa should prevent thrombus formation without prejudice to hemostasis and platelet function maintaining normal thrombin level. Moreover it was found that inhibitors of factor Xa have a broad therapeutic window between adverse hemorrhage and thrombosis [7, 8]. Orally available factor Xa inhibitors are expected to become the most important antithrombotic drugs and be able to overcome all the disadvantages connected with the current treatment.

At the present time there are several chemical compounds that are direct factor Xa inhibitors. However a part of them has not passed clinical trials (Otamixaban [9], Darexaban [10]). Another part of the factor Xa inhibitors is now at the stage of clinical trials (Betrixaban [11]), and only three compounds have been just approved for clinical practice (Rivaroxaban [12], at the end of 2011; Apixaban [13], in 2014; and Edoxaban [14], in 2015). However, there are references about side effects and contraindications of these drugs; for example, Rivaroxaban is contraindicated in hepatic disease patients and it is not recommended in patients with creatinine clearance of <15 mL/min; also Apixaban and Edoxaban inhibit hepatic functions and have other contraindications [3, 15]. In addition there are data about problems with bioavailability and toxicology of candidate compounds [2, 16], which means that the development of new low molecular weight factor Xa inhibitors is still the problem of vital importance. Furthermore, development of efficient tools for computer aided structural based drug design, especially modeling of proteins interaction with low molecular weight ligands for virtual screening of chemical compounds databases and for selection of molecules candidates to become inhibitors of a given target protein, is of particular importance.

In this paper a SOL program [17, 18] and a DISCORE program [19] are used as the molecular modeling tools for docking and postdocking optimization (or “postprocessing”), respectively. Off-the-shelf compounds databases virtual screening has been carried out by these programs. The compounds showed that the best values of protein-ligand binding energy in computer modeling have been ordered, received, and tested experimentally* in vitro*. As a result, new inhibitors of factor Xa have been identified, and a new candidate compound has been designed, synthesized, and tested* in vitro*. This new compound has demonstrated inhibition activity of factor Xa with IC_50_ = 0.7 *μ*M.

## 2. Materials and Methods of the Research

### 2.1. Molecular Model of Protein

**Figure 1:**
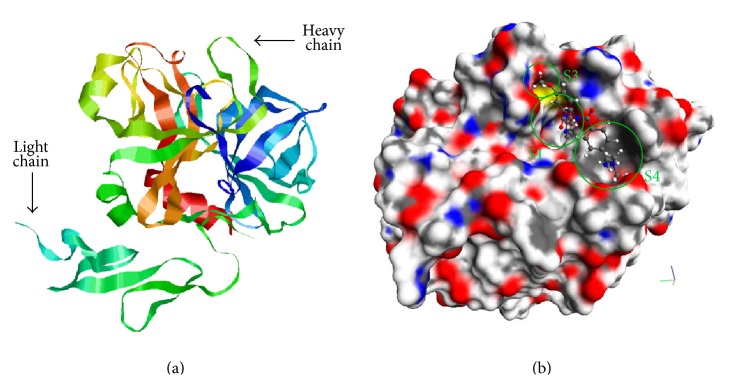
(a) Structure of factor Xa (molecular editor RasMol, Protein Data Bank ID of the complex is 3IIT). (b) Betrixaban molecule in the active site of factor Xa. The binding pockets are indicated by green circles (molecular editor MolRed).

Factor Xa is a serine protease protein with weight of about 45 kDa. It consists of light (139 residues) and heavy (303 residues) chains linked by a disulfide bridge (Figure 1(a)). The heavy chain includes characteristic for serine protease domain with trypsin-like structure in the form of *β*-barrel with catalytic triad Ser195-His57-Asp102. Substrate specificity of factor Xa recognizes Ile-Glu/Asp-Gly-Arg sequence and hydrolyzes the bond after the arginine residue [20].

The binding site is divided into 4 pockets: S1, S2, S3 (S1-*β* in some works), and S4 [21–23]. The respective moieties of inhibitors are designated as Р1, Р2, Р3, and Р4 depending on the pocket of the enzyme binding site where the moiety is located. S1 pocket is the most important pocket for binding and it determines the protein-ligand binding energy. It is a negatively charged deep groove formed by such amino acids like Asp189, Ser195, and Tyr228 that serves for binding of the native substrate arginine. Strong binding of the positively charged main inhibitor fragment P1 occurs in this pocket. S4 pocket binding isoleucine of the thrombin sequence is formed by Tyr99, Phe174, Trp215 residues and it is responsible for hydrophobic and stacking ligand-protein interactions. In contrast with thrombin, S2 pocket of factor Xa is ill-defined and small and merges with other pockets. It is considered that S2 pocket is structurally formed by Gly216 and Gly218, but many authors do not prefer to distinguish this place but to attribute it to S1 or S4 pockets. (S1-*β*) pocket is located in the edge of S1 pocket and it is able to form specific hydrogen bonds with the ligand (Figure 1) [24, 25].

Factor Xa three-dimensional structures were taken from Protein Data Bank [26]. In the course of the work 64 structures of the protein have been prepared. These 64 complexes were selected according to the following criteria: source organism was “Homo sapiens,” X-ray resolution was better than 3 Å, all these structures have ligands, and the binding affinities were known for the ligands.

Preparation of the structures includes the following:

1. Remove water being used for crystallization as well as other molecules unrelated to the structure of the protein, for example, ligands and sugars and salts molecules.
2. Check the protein structure for unresolved amino acid residues or atoms. If there are such species their restoration has been carried out with the help of the Superimpose program, developed by Dimonta, Ltd., which carries out a superposition of two protein structures on one another. Superimpose program performs search for such a transformation containing translation and rotation of the reference protein as a rigid body that minimizes the root mean square deviation (RMSD) between the positions of all heavy (nonhydrogen) atoms of two protein structures. The protein structure without missed residues or atoms is used as the reference one and the protein structure with missed residues and/or atoms is used as the second structure for the Superimpose program input. After the execution of Superimpose program the coordinates of missed atoms are transferred from the reference structure into the defective one and the latter is restored.
3. As much as there is usually no information about positions of light (hydrogen) atoms in such crystalline protein structures, one needs to supplement such structures with hydrogen atoms before the protein atoms typification and the generation of the protein-ligand interaction potentials. Adding the hydrogen atoms to the structure has been carried out by the Aplite program [27], developed by Dimonta, Ltd. This program adds hydrogen atoms to the protein structure according to standard protonation states of amino acids at pH = 7.2–7.4, that is, under normal physiological conditions. Histidine protonation state is selected according to the electrochemical potentials of the surrounding atoms. After the prearrangement of the hydrogen atoms the local energy optimization in the frame of the MMFF94 [28] force field was carried out with respect to positions of all hydrogen atoms. For torsionally labile heavy atoms with hydrogen atoms (e.g., the hydroxyl in tyrosine) all possible torsional rotations with some step were checked during the optimization. However, the optimization of proteins heavy atoms was not carried out. The Aplite program also performs typification of all protein atoms in the frame of MMMF94 force field for further docking procedure.
4. Stereochemical evaluation of the protein model was fulfilled by PROCHECK program [29]. According to the plotted Ramachandran diagram representing a distribution of torsion angles (rotations around C*α*–N and C*α*–CO bonds) the vast majority of residues (91%) are located in the most preferable conformational regions.

### 2.2. Docking Program SOL

The original SOL and SOLGRID programs were used for docking [17–19]. The SOL program demonstrates [19, 30] good and excellent (for different target proteins) ability to select active ligands from a mixture of active and inactive ones, as well as accurate positioning of native ligands in the active site of different target proteins. SOL is not worse than the widely used AutoDock 3.05 program [31] and sometimes SOL is even better. Moreover the SOL program was well proven in CSAR 2011-CSAR 2012 testing [19], wining one of the first places in the competition.

The SOL program allows calculating the protein-ligand binding energy using the genetic algorithm for the global protein-ligand energy minimum search, taking into account all interactions in the frame of the MMFF94 force field [28]. The protein active site (with the exact coordinates of all its constituent atoms) is represented by a set of energy grids describing various types of protein-ligand interactions. The energy grids are built by the SOLGRID program to expedite subsequent docking, wherein the protein is considered as a rigid structure and the ligand is flexible at the expense of all possible intrinsic torsions and translation-rotation transformations as a rigid body. A distinctive feature of the SOL program is taking into account desolvation effects within a simplified version of the generalized Born model [32].

The SOL program has been successfully used to develop new inhibitors of thrombin [33] and urokinase [34]. Before virtual screening of ligand databases docking of native ligands and/or ligands with known binding energies into the factor Xa active site was carried out. Experimental data on the binding constants were taken from the PDB database and from the articles [35–39]. Several charge states in accordance with the calculations of pKa by MARVIN program were considered when preparing the ligands [40].

### 2.3. Postdocking Optimization Program DISCORE

The original DISCORE program has been used [19] to improve the prediction of binding energy. The DISCORE program carries out the local optimization of a ligand in the protein by L-BFGS method [41] in solvent for the rigid protein or taking into account mobility of some protein atoms, using the docked ligand position as the initial position and using the complete typification of atoms within the MMFF94 force field. In this case, the solvent can be taken into account within the framework of one of the three types of continuum solvent model: Surface Generalized Born (SGB), COnductor-like Screening MOdel (COSMO), and Polarizable Continuum Model (PCM) [42, 43]. Also the program calculates the refined protein-ligand binding energy taking into account the elimination of the simplifications that have been used in the SOL docking program, including calculation of the protein, the ligand, and their complex interactions with solvent and estimation of the entropy component of the binding free energy more accurately.

The DISCORE program represents the energy of the protein-ligand interaction (the scoring function) in terms of linear combination of its components: the energy of the direct Coulomb interaction between the ligand and protein atoms charges (Δ*G*_Coulomb_), the energy of the direct Van der Waals interactions (Δ*G*_VDW_) between the ligand and protein atoms, polar (Δ*G*_pol_) and nonpolar (Δ*G*_np_) contributions to the desolvation energy, the ligand strain energy (Δ*G*_LS_), and the entropy contribution:

(1) ΔGbind=k1ΔGCoulomb+k2ΔGVdW+k3ΔGpol+k4ΔGnp+k5ΔGLS+k6kNTORS+k7ΔGtr,

where *kN*_TORS_ is the binding entropy contribution caused by freezing of the ligand torsion degrees of freedom after protein-ligand binding and the term Δ*G*_tr_ is caused by freezing the degrees of freedom responsible for the rotation and translation of the ligand as a whole rigid body [44]. The dimensionless coefficients *k*_*i*_ are fitted to reproduce well experimental data on inhibition constants for a training set of protein-ligand complexes.

### 2.4. Database of Screenings Compounds

The following procedure is often used at the first step for new inhibitors development: one needs to choose a database of drug-like compounds that have been already synthesized and are available for the order and then to perform virtual screening of this database, that is, to analyze and select the promising compounds using molecular modeling methods (docking and postdocking optimization) with the subsequent order and experimental testing of the selected compounds on inhibition of the given target protein. Several practical goals could be achieved in such investigation. Firstly, it allows identifying new molecular groups providing main contribution to new inhibitors binding with the target protein. Secondly, it results in the discovery of new application of well-known compounds and, finally, it helps to find new compounds that can become basis for further development of new patentable inhibitors.

During the present research screening of two compounds libraries has been carried out: NCI Diversity (USA) database [45] containing 1888 compounds interesting for docking in factor Xa compounds and the library of Voronezh State University (the department of organic chemistry), containing 14271 compounds. The compounds structures have been prepared (charge states of molecules were defined and hydrogens were added) by the Open Babel [46] and MARVIN programs.

### 2.5. Materials for Experimental Testing

Human factor Xa was obtained from Hematologic Technologies Inc. (USA). The Xa-specific chromogenic substrate S2765 (Z-D-Arg-Gly-Arg-pNA·2HCl) was purchased from Chromogenix (USA). All other chemicals were of reagent and analytical grade.

### 2.6. Method of Experimental Testing

The kinetics of factor Xa inhibition was determined from the hydrolysis reaction of a specific substrate by the enzyme in the presence of tested substances. The chromogenic substrate S2765 (Z-D-Arg-Gly-Arg-pNA·2HCl, Chromogenix, Instrumentation Laboratory Company, Lexington, MA 02421, USA) was used for registering accumulation of* p*-nitroaniline as a colored product by spectrophotometer.

Plate wells were filled with 20 mM HEPES (pH 8.0) containing 140 mМ NaCl and 0.1% polyethylene glycol (molecular weight 6000 Da). Thereafter, the substrate was sequentially added to each well (final concentration, 250 *μ*M), followed by the substance being tested (to a final concentration that was varied) and Xa (final concentration, 0.5 nM). The hydrolysis rate was monitored spectrophotometrically at 405 nm (absorption maximum of the reaction product* p*-nitroaniline). The results of these measurements were implied to measure the kinetics of factor Xa inhibition by various inhibitors.

The initial rate was determined as the slope of the linear part of the kinetic curve over the first 10 to 20 min of measurement. The inhibitory effect was expressed as the percentage of reduction of the initial hydrolysis rate. The reaction rate in the absence of any inhibitor was taken as 100%. Each result is the mean of two parallel determinations. The inhibitor concentration was determined decreasing hydrolysis rate to 50% (IC_50_). Processing of results was performed using the standard graphics Origin 6.0 program (Microcal Software Inc., MA, USA). Graphs of optical density against time were plotted.

### 2.7. Synthesis of the Compound **17f**

**Scheme 1:**
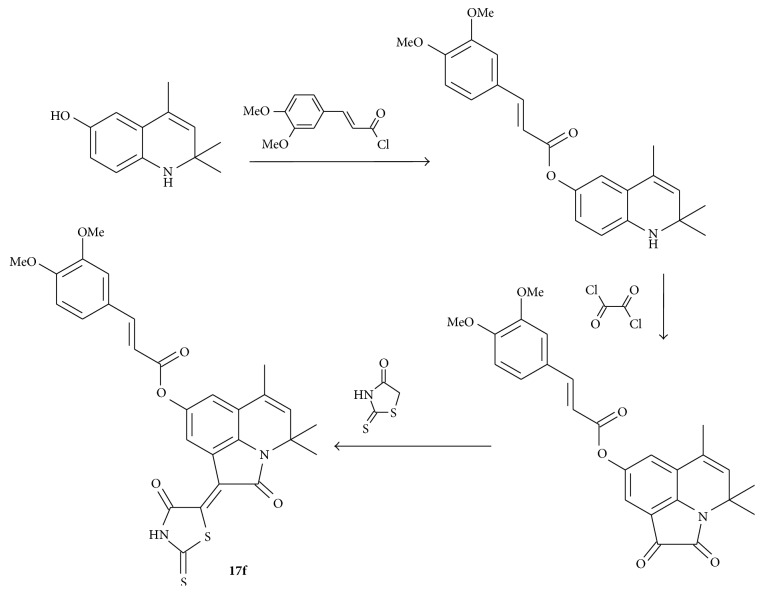


The compound** 17f** (4,4,6-trimethyl-2-oxo-1-(4-oxo-2-thioxo-1,3-thiazolidin-5-ylidene)-1,2-dihydro-4*H*-pyrrolo[3,2,1-*ij*]quinolin-8-yl-3-[3,4-bis(methyloxy)phenyl]prop-2-enoate) was synthesized by known methods from 6-hydroxy-2,2,4-trimethyl-1,2-dihydroquinoline [47] consistently carrying out the acylation reaction at the hydroxyl group by 3,4-dimethoxy-cinnamic acid chloride [48] annelation of pyrrole-1,2-dione fragment on the* Stolle *reaction by the action of oxalyl chloride [49] and subsequent condensation on the *β*-carbonyl group of annelated fragment with rhodanine [50] (Scheme 1).

^1^Н NMR spectra were recorded on a Bruker DRX-500 instrument (500 MHz) in DMSO-d_6_. The residual solvent protons were used as reference in ^1^Н NMR spectra (*δ*_H_ 2.50 ppm). The mass spectra with EI ionization were recorded on an MX-1321 mass spectrometer with a direct introduction of sample at 100–150°C and the accelerating voltage equal to 70 eV. The elemental analysis was performed on a PerkinElmer 2400 instrument. Melting points were determined on a PTP-M apparatus. The reaction progress and the purity of the obtained compounds were controlled by TLC on Silufol UV-254 plates, eluent 10 : 1 EtOAc/MeOH.

The starting compound was synthesized according to a published method [47–49]. Commercially available reagents from Lancaster also were used in the syntheses.

### 2.8. 4,4,6-Trimethyl-2-oxo-1-(4-oxo-2-thioxo-1,3-thiazolidin-5-ylidene)-1,2-dihydro-4*H*-pyrrolo[3,2,1-*ij*]quinolin-8-yl-3-[3,4-bis(methyloxy)phenyl]prop-2-enoate **17f**

The mixture of starting 4,4,6-trimethyl-1,2-dioxo-1,2-dihydro-4*H*-pyrrolo[3,2,1-*ij*]quinolin-8-yl-3-[3,4-bis(methyloxy)phenyl]prop-2-enoate (0.433 g, 1 mmol) and rhodanine (0.133 g, 1 mmol) in butanol-1 was refluxed for 2 hours. The initially red solution became dark brown during this process, and a precipitate started to form. The dark powdery precipitate that formed after cooling was filtered off, washed with water and alcohol, and dried. The obtained compound was not recrystallized. Yield − 0.51 g (93%). M.p. 280–282°С. The NMR ^1^H spectrum, *δ*, ppm: 1.65 (s, 6H, С(CH_3_)_2_); 1.99 (d,* J* = 1.3 Hz, 3Н, CH_3_); 3.82 (s, 3H, OCH_3_); 3.83 (s, 3H, OCH_3_); 5.56 (d,* J* = 1.4 Hz, 1H, CH); 6.80 (d,* J* = 15.9 Hz, 1H, CH=CHCO); 7.02 (d,* J* = 8.4 Hz, 1H, H-Ar); 7.11 (d,* J* = 2.2 Hz, 1H, H-Ar); 7.332 (dd,* J* = 8.4, 2.0 Hz, 1H, H-Ar); 7.45 (d,* J* = 2.0 Hz, 1H, H-Ar); 7.81 (d,* J* = 15.9 Hz, 1H, CH=CHCO); 8.28 (d,* J* = 2.1 Hz, 1H, H-Ar); 14.08 (br s, 1H, NH);. MS: m/e (%) 548 [М+] (3), 358 (7), 343 (6), 255 (3), 227 (2), 199 (14), 191 (100), 163 (12). Found, % С 61.41; H 4.44; N 4.95; S 11.82. C_28_H_24_N_2_O_6_S_2_. Calculated, % С 61.30; H 4.41; N 5.11; S 11.69.

## 3. Results and Discussion

### 3.1. Results of SOL and DISCORE Programs Validation

#### 3.1.1. Validation of SOL Program

The positioning quality of the docking by the SOL program was evaluated on 64 protein-ligand complexes taken from PDB database. The root mean square deviations (RMSD) between native ligand conformations (the ligand poses taken from PDB) and docked ones were calculated. The data were averaged over all atoms of the ligand. It was found that RMSD between native and docked poses for 94% complexes were less than 2 Å. This docking quality is considered usually as “good” one.

**Figure 2:**
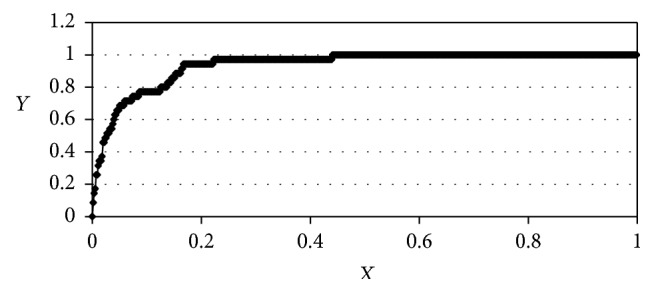
Enrichment curve for docked ligands of factor Xa. AUC = 0.94. We denote the total number of the ligands ranged by scoring function as *N* (total), the total number of the active compounds as *N* (total active), the top *N* ligands from this ranged list of the ligands as *N*, and the number of active ligands from these top *N* ligands as *N* (active). Then *X* = *N*/*N* (total) and *Y* = *N* (active)/*N* (total active).

Evaluation of the discriminatory ability of the model has been carried out using the “enrichment curve” [51] method. Area under the enrichment curve (AUC) can be interpreted as a probability that active compounds will be above inactive ones in the top-scoring list. Since 64 selected PDB complexes have binding affinities in PDB database, they were accepted as active inhibitors. The enrichment curve for the ligands of factor Xa docked by the SOL program has been plotted on the basis of factor Xa active inhibitors and inactive compounds from NCI Diversity database (Figure 2). The AUC value for the factor Xa obtained by SOL program is equal to 0.940.

For further virtual screening the factor Xa structure from PDB ID 3IIT complex has been chosen. It was done on the basis of analysis of all prepared 64 PDB factor Xa complexes structures due to several reasons as follows:

(1) Resolution 1.80 Å.
(2) *R*-factor = 0.203 (this value shows how well a particular model structure corresponds to the observed electron density; *R* < 0.25 is considered as “good” value of *R*-factor) [51].
(3) Absence of unresolved amino acid residues in the main chain.
(4) Presence of missing atoms only in amino acid residues that are far from the active site.
(5) Good stereochemical quality of the protein structure according to its Ramachandran plot.
(6) Docking of the native ligand from the 3IIT complex giving good results: RMSD between the docked ligand pose and the native one is 0.601 Å and the SOL scoring function is −7.33 kcal/mol. 50 independent runs of SOL program with 3IIT complex produce the ligand poses which can be gathered in one cluster with RMSD between these poses of the ligand being less than 1 Å.

#### 3.1.2. Validation of DISCORE Program

The procedure of coefficients *k*_*i*_ fitting in the equation for Δ*G*_bind_ (see Section 2.3) for factor Xa target protein was carried out to improve inhibitor prediction quality of the DISCORE program.

For this purpose all found inhibitors and candidates to the factor Xa inhibitors were divided into 2 sets, namely, training and testing ones. The training set contains 26 molecules having good inhibition constants and 21 ones, in which inhibition constants were above 1000 *μ*M in experiments. The test set consists of 38 inhibitors of factor Xa with good binding energies and 26 molecules that have not shown binding to this enzyme in experiments. Molecules having good inhibition constants were taken from selected 64 PDB complexes. The experimental data (inhibition constants) for active and inactive ligands were collected from PDB and [35–39]. Molecules were distributed among the sets in a random way.

It should be noticed that despite good positioning quality the docking program is not always able to distinguish good inhibitors from excellent ones and bad inhibitors from inactive compounds and consequently the false-positive and false-negative results appear in the virtual screening process. The application of the postdocking optimization makes it possible to improve significantly the accuracy of binding energy predictions compared with the estimate given by the SOL docking program.

It should also be emphasized that the SOL program produces a rather small range of binding energies (the scoring function values) in the calculation; for example, for factor Xa the absolute values of binding energies are within the range from 2.7 to 8.8 kcal/mol for all compounds from the training and testing sets. For this reason, it is very difficult to define a boundary energy separating active and inactive compounds without false predictions. The DISCORE program gives a greater range of binding energies using *k*_*i*_ coefficients found in the fitting procedure. Furthermore, the SOL program makes some incorrect predictions due to the simplified scheme of protein-ligand binding energy calculations mainly due to the absence of the local energy optimization and too simplified calculation of the desolvation energy when nonlocal molecules interactions with solvent continuum are approximated by the respective local energy grid. The application of the DISCORE postdocking optimization after docking can improve the results due to the local energy optimization of the docked poses and the accurate calculation of the desolvation energy in the frame of one of the implemented implicit solvent models (SGB, PCM, and COSMO) and due to binding energy tuning with the fitting coefficients *k*_*i*_ specially adapted to the factor Xa (Table 1).

According to the DISCORE results the boundary energy of −6.5 kcal/mol has been chosen to divide active and inactive compounds. Additional SOL program calculations for molecules from the training and testing sets show that the energy of −7 kcal/mol sifts out all inactive compounds; that is, SOL binding energies of all inactive ligands and ones of several active inhibitors lie above this boundary. Therefore compounds with SOL binding energies which were more negative than −7 kcal/mol were primarily subjected to postdocking optimization by the DISCORE program in the big libraries screening.

### 3.2. Docking of the NCI Database

Docking of 1888 compounds from the NCI Diversity database has been carried out by the SOL program. This screening gives only 6 compounds for which the SOL binding energy was better than −7 kcal/mol (i.e., it was more negative than −7 kcal/mol). Also SOL binding energies were below −6.5 kcal/mol for another 74 compounds. The only two compounds have been identified (see Table 2) having simultaneously high negative SOL and DISCORE binding energies after postdocking optimization of all these 74 compounds (including those 6 compounds with SOL binding energy below −7 kcal/mol).

Among the ligands from the NCI Diversity library no compounds bound with the S4 pocket have been identified, but all of them have P1 positively charged moiety that provides binding interactions with the negatively charged aspartate in the S1 pocket. Also a large contribution of Coulomb interactions in the protein-ligand binding energy is a distinctive feature of such compounds. Similar features are presented in some known factor Xa inhibitors. For example, benzamidine moiety interacting with S1 pocket (as well as NSC357777 compound) is also a part of Otamixaban inhibitor and this moiety is typical not only for factor Xa inhibitors but also for inhibitors of other serine proteases. Thus, two compounds from the NCI Diversity database can be considered as candidates to become inhibitors of factor Xa.

These compounds have not been ordered for experimental verification.

### 3.3. Docking of Voronezh State University Database

Structures of potential inhibitors from the Voronezh ligand library are in stark contrast to ones found in the NCI Diversity. Most of the compounds from the Voronezh library have no charge at the P1 moiety but have large hydrophobic aromatic P4 moieties. Accordingly, the reverse pattern of binding was observed for these compounds: the main feature of their binding is the interaction with aromatic amino acids in the S4 pocket.

One of the compounds that showed the most negative calculated binding energy is VGY-0018989. It has the conjugated aromatic rings system which is positioned parallel to Trp215 after docking and such a configuration most likely indicates that the stacking interactions are presented.

Another interesting ligand is VGY-0101011. It consists of phenyl bromobenzene as a P4 moiety. The introduction of a halogen in the aromatic ring is supposed to be beneficial to stacking interactions due to the appearance of electron density deficit and it leads to increase of potential inhibitor affinity. For example, it has been proved experimentally [52] that substitution of chlorine atom instead of iodine one in this position leads to two-order reduction of the inhibition constant. It is interesting to note that such substituent benzene moieties provide the optimal geometry for binding in the S4 pocket of factor Xa because more bulky substituents have lower binding energy. This agrees well with the experimental data obtained in the study of similar ligands [53].

Other ligands containing aromatic rings or ring systems also can bind with Tyr99 and Phe174 or with Trp215 depending on their structures. Also in some cases the probability of T-stacking cannot be excluded, when hydrogen atoms with partially positive charges of one aromatic system are directed to the center of another aromatic ring perpendicular to the plane of the latter.

It should be noted that stacking is a quite controversial type of interactions and although it is defined as aromatic (or *π*-*π*) noncovalent interaction between organic compounds, containing aromatic components, its exact nature is a subject of discussions [54].

It is possible that the stacking interactions caused the unexpected results of the DISCORE postdocking optimization: values of the DISCORE scoring function are in the range between −0.93 and −4.67 kcal/mol for all compounds chosen by the SOL docking procedure. For all interesting molecules from the Voronezh State University Database of organic compounds the Coulomb component of the binding energy is very small comparing with this term for other ligands taken from the NCI Diversity set or from the learning and testing sets used for fitting *k*_*i*_ coefficients in regression of Δ*G*_bind_ in the DISCORE program. As a result, the total binding energy (the DISCORE scoring function) has also low negative values. However, it seems that DISCORE (as well as SOL) does not take into account stacking interaction due to peculiarities of the MMFF94 force field. Furthermore the learning and testing sets of ligands do not contain examples of molecules which can realize stacking interactions with factor Xa active site.

The interesting nontypical result is that neutral moieties of majority of compounds from the Voronezh database enter into S1 pocket containing the negatively charged aspartate. This can be explained as follows. These ligand neutral moieties as a rule are the pyridine or piperidine analogs with positive partial charges of carbon atoms and they are attracted by the negatively charged aspartate.

### 3.4. Experimental Validation of Compounds from Voronezh State University Database

According to the results of our molecular modeling with SOL and DISCORE programs 17 compounds have been selected and investigated in the frame of* in vitro* experiments. The most of these compounds inhibited the hydrolysis of the specific substrate by factor Xa in a dose-dependent manner (Figure 1). Some compounds were poorly soluble; therefore, these compounds could not reach 100% inhibition of factor Xa, and some compounds could not reach 50% inhibition (see Table 3, weakly inhibited). Rivaroxaban scoring function and IC_50_ measured with the same conditions are also given in Table 3.

Four compounds (VGY-0160076, VGY-0017263, VGY-0015091, and VGY-0039772) out of 17 tested compounds did not show any inhibitory activity. One compound (VGY-0038035) weakly inhibited factor Xa (at concentration of 51 *μ*M the only 34% inhibition has been measured); larger inhibition was not achieved due to the poor solubility of this compound. The remaining compounds showed inhibitory activity with IC_50_ = 1.8–40 *μ*M (Table 3).

Thus, we can say that two main groups of factor Xa inhibitors with micromolar activity have been identified (according to the type of the heterocyclic matrix): pyrroloquinolines (VGY-0018989, VGY-0018521, VGY-0041455, and VGY-0027889) and quinazolines, containing in the 2 positions an amino group, included in or associated with the heterocyclic fragment (VGY-0018863, VGY-0035262, VGY-0006889, VGY-0037900, VGY-0163641, and VGY-0037972). All measured compounds are inferior to Rivaroxaban with respect to IC_50_ value. However, all these novel compounds were designed relying solely on the docking results and they may be used as the basis for further development to increase activity of new synthesized compound, for example, by their solubility improvement.

### 3.5. Experimental Verification of the New Compound

On the basis of the lead compound VGY-0018989 analysis another one has been suggested for synthesis,** 17f** (Table 4). In contrast to the VGY-0018989 the** 17f** compound does not bear negative partial charges at P1 fragment, which should provide better interaction with the negatively charged aspartate of the active site. The synthesis scheme of this compound has been given in Methods.

**Figure 3:**
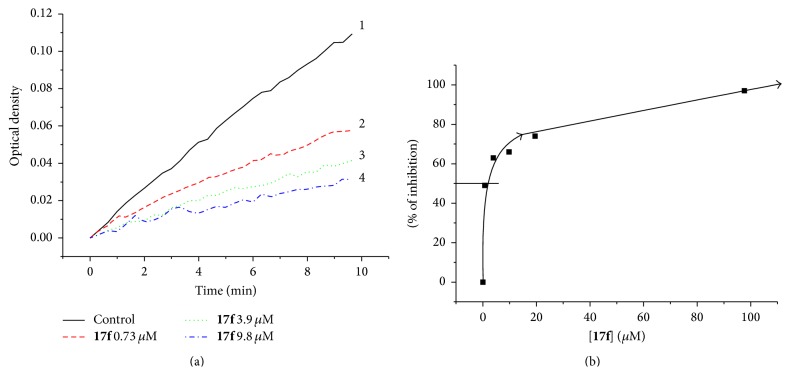
The results of experimental testing of** 17f** compound. (a) Inhibition of factor Xa-induced chromogenic substrate hydrolysis in buffer systems by different concentrations of** 17f**. (b) The dependence of inhibition of factor Xa-induced chromogenic substrate hydrolysis on the inhibitor concentration.

The kinetics of hydrolysis of the specific substrate by factor Xa in the buffer solution and in the presence of various concentrations of** 17f** compound was measured. The results are presented in Figure 3.

## 4. Conclusion

The results of molecular modeling tools application for new micromolar inhibitors of coagulation factor Xa identification in two databases of ready-made organic compounds, design, synthesis, and experimental testing of the new factor Xa inhibitors are presented in this work. The protein 3D structural model was created on the basis of the factor Xa protein-ligand structures from the Protein Data Bank. Validation of the SOL docking program demonstrated high quality of factor Xa native ligands positioning (RMSD between native and docked poses was less than 2 Å for 94% complexes out of the 64 protein-ligand complexes taken from PDB database) as well as high performance of the identification of known factor Xa inhibitors from a large set of inactive compounds based on their ranking by the calculated protein-ligand binding energies (the enrichment plot AUC = 0.94).

Methodology of the molecular-mechanical postdocking optimization for protein-ligand binding energy refinement after docking in the frame of the DISCORE program was applied to factor Xa. It was shown that the number of false predictions is reduced significantly by the postdocking optimization. This result demonstrates that successive application of SOL docking and DISCORE postdocking optimization programs can noticeably improve reliability of new inhibitor predictions. The virtual screening of two ready-made organic compounds libraries, the NCI Diversity (USA) [45] and the database of organic chemistry department of Voronezh State University (Russia), was carried out, and several micromolar inhibitors of factor Xa have been identified and their activities were proved experimentally* in vitro*. On the basis of the modeling and experimental results the new factor Xa inhibitor has been synthesized and tested* in vitro*.

The outcomes of the present research show high efficiency of the SOL docking program used for the virtual screening of large databases containing low molecular weight compounds in order to find inhibitors for a given target protein: 12 compounds of 17 selected ones demonstrated inhibition activity of factor Xa at the micromolar level. It should be noted that four of them show relatively high inhibitory activity with IC_50_ = 1.8–3.1 *μ*M: VGY-0018989 (4,4,6-trimethyl-2-oxo-1-(4-oxo-2-thioxo-1,3-thiazolidin-5-ylidene)-1,2-dihydro-4*H*-pyrrolo[3,2,1-*ij*]quinolin-8-yl-3-(3-nitrophenyl)prop-2-enoate, VGY-0101011 (8-(4-bromophenyl)-*N*-(3-methoxyphenyl)-4,7-dimethylpyrazolo[5,1-*c*][1,2,4]triazine-3-carboxamide), VGY-0006889 (6-[(1,3-benzothiazol-2-ylthio)methyl]-2-[(5,6-dimethylquinazolin-2-yl)amino]pyrimidin-4(3*H*)-one), and VGY-0035262 (7-(4-methoxyphenyl)-2-[(4,6,7-trimethylquinazolin-2-yl)amino]-7,8-dihydroquinazolin-5(6*H*)-one).

The synthesized compound** 17f** (4,4,6-trimethyl-2-oxo-1-(4-oxo-2-thioxo-1,3-thiazolidin-5-ylidene)-1,2-dihydro-4*H*-pyrrolo[3,2,1-*ij*]quinolin-8-yl-3-[3,4-bis(methyloxy)phenyl]prop-2-enoate) shows excellent inhibitory activity with IC_50_ = 0.7 *μ*M. Further optimization of the new inhibitors may lead to a new class of anticoagulants.

## Figures and Tables

**Table 1:** The docking and postdocking optimization results for the factor Xa test set of ligands.

After SOL	After DISCORE
3 false-positive ligands	0 false-positive ligands
1 false-negative ligand	0 false-negative ligands
23 true-negative ligands	26 true-negative ligands
37 true-positive ligands	38 true-positive ligands

**Table 2:** NCI IDs, structures, SOL, and DISCORE scoring functions of lead compounds from the NCI Diversity database.

NCI ID	Structure	SOL score, kcal/mol	DISCORE score, kcal/mol
NSC357777	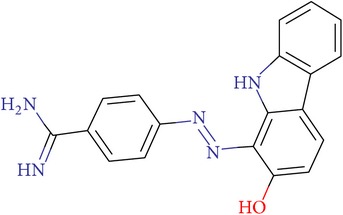	−7.45	−8.82

NSC44677	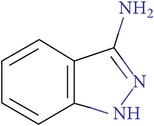	−7.06	−7.30

**Table 3:** IDs, structures, SOL scoring function values, and results of experimental measurements of IC_50_ values of factor Xa inhibition for various compounds.

ID	Structure	SOL score, kcal/mol	Inhibition of hydrolysis rate specific to the factor Xa substrate in the buffer system IC_50_, *μ*M
VGY-0018989	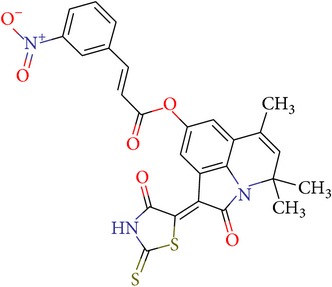	−7,46	1,8

VGY-0018863	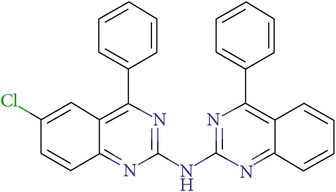	−7,27	10

VGY-0018521	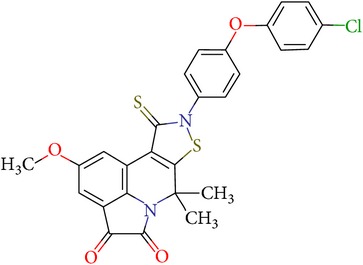	−7,04	6,3

VGY-0035262	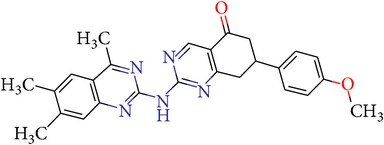	−7,29	3,1

VGY-0160076	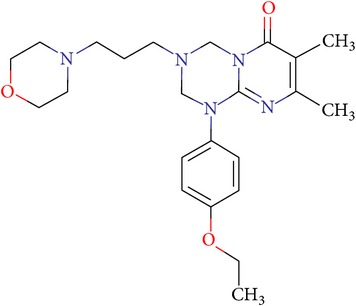	−7,02	Not inhibited

VGY-0038035	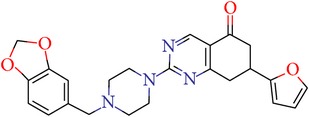	−7,27	Weakly inhibited

VGY-0006889	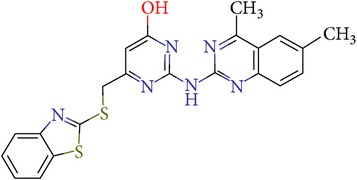	−7,09	2,3

VGY-0037972	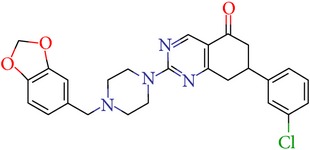	−7,38	5

VGY-0101011	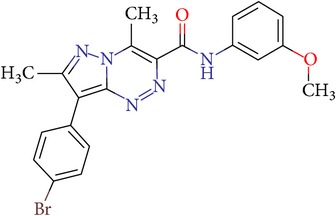	−7,12	2

VGY-0037900	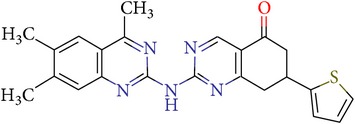	−7,07	10

VGY-0041455	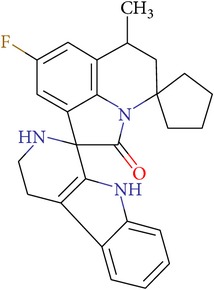	−7,12	40

VGY-0016013	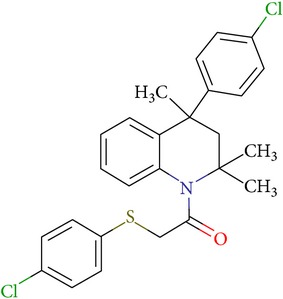	−7,04	19

VGY-0027889	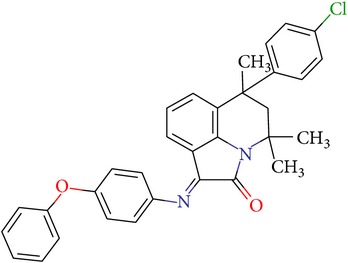	−7,03	20,6

VGY-0017263	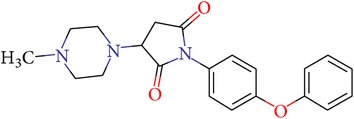	−7,43	Not inhibited

VGY-0015091	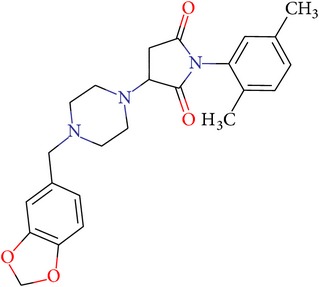	−7,26	Not inhibited

VGY-0039772	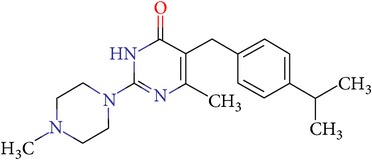	−7,17	Not inhibited

VGY-0163641	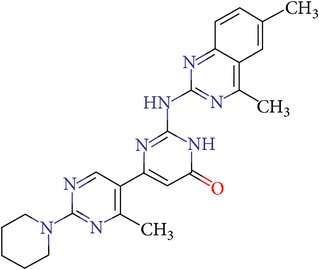	−7,17	12

Rivaroxaban	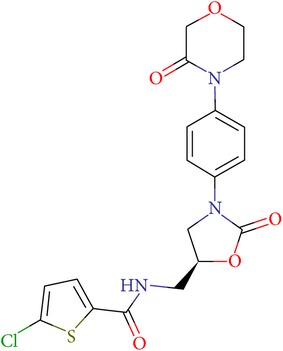	−7,20	0,21 nM

**Table 4:** ID, structure, SOL scoring function, and results of experimental measurements of factor Xa activity in the presence of **17f** compound.

ID	Structure	SOL score, kcal/mol	DISCORE score, kcal/mol	Activity of factor Xa in the presence of the inhibitor, IC_50_, mcM
**17f**	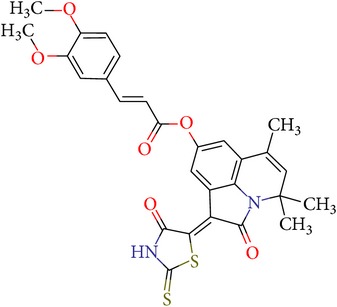	−7,05	−3,04	0,7
